# The association of Red cell distribution width and in-hospital mortality in older adults admitted to the emergency department

**DOI:** 10.1186/s13049-016-0274-8

**Published:** 2016-06-06

**Authors:** Soo Hyun Kim, Jeong Hoon Yeon, Kyu Nam Park, Sang Hoon Oh, Seung Pill Choi, Young Min Kim, Han Joon Kim, Chun Song Youn

**Affiliations:** Department of Emergency Medicine, Seoul St. Mary’s Hospital, #505 Banpo-Dong, Seocho-Gu, Seoul, 137-701 Republic of Korea

**Keywords:** Mortality, Older adults, Erythrocyte indices

## Abstract

**Background:**

The objective of the study was to test the hypothesis that elevated red cell distribution width (RDW) at admission increases the risk of mortality in older patients admitted to the emergency department (ED).

**Methods:**

We performed a retrospective analysis of patients admitted to the ED between May 2013 and October 2013. We included patients who were older than 65 years who visited the ED with any medical problems. Baseline RDW values were measured at the time of admission to the ED. The primary outcome was all-cause in-hospital mortality. Multivariate logistic analysis was performed.

**Results:**

A total of 1,990 patients were finally included in this study. The mean age was 75 years (SD 7), and 936 (47 %) subjects were male. The in-hospital mortality rate was 3.76 % (74 patients). RDW values higher in non-survivors than in survivors (15.9 ± 2.5 vs. 13.8 ± 1.7, *p* < 0.001). Multivariate logistic analysis showed that RDW was associated with all-cause in-hospital mortality after adjusting for other confounding factors.

**Discussion:**

RDW value at admission is an independent predictor of all-cause in-hospital mortality among patients older than 65 years. After adjustment for multiple confounders, the all-cause in-hospital mortality rate increased by 21.8% for each 1% increase in RDW.

**Conclusion:**

These results show that RDW at admission is associated with in-hospital mortality among patients older than 65. Thus, RDW at admission may represent a surrogate marker of disease severity. We caution against using these findings to aid clinical decision-making process until they are externally validated.

## Background

The proportion of elderly individuals in the population is increasing, and older people visit the emergency department (ED) more frequently than younger adults [1–4]. Older patients often present with atypical signs and symptoms and have multiple comorbidities that complicate accurate diagnosis and treatment [5]. Furthermore, these patients have a higher level of acuity, higher risk for hospitalization and higher intensive care unit (ICU) admission rate [6–9]. However, there are few studies measuring the association between the risk factors and in-hospital mortality in older patients admitted to the ED.

Red cell distribution width (RDW) is a quantitative measure of variability in the size of circulating erythrocytes and is routinely reported to physicians in clinical practice as part of the automated complete blood count (CBC). RDW is used as an ancillary test to help diagnose different types of anemia. Recent studies have shown that higher RDW is associated with increased mortality risk in different clinical settings such as clinically significant cardiovascular disease, stroke, septic shock, bacteremia and community-acquired pneumonia [10–15]. Although the exact mechanisms that underlie the association between RDW and mortality are unknown, high RDW may have an association with the presence of an ongoing disease process, such as inflammation, tissue hypoperfusion, oxidative stress, or renal failure [16]. RDW is known to be a strong predictor of mortality in the general population of middle-aged and older adults [16, 17] and is also considered an age-associated prognostic biomarker in adults aged 45 and older [17]. However, the prognostic value of RDW in older patients admitted to the ED has rarely been investigated. In addition, older adults admitted to the ED are likely to have multiple comorbidities.

We tested the hypothesis that elevated RDW at admission increases the risk of mortality in older patients admitted to the ED. We assessed the association between RDW at admission and in-hospital mortality in older patients admitted to the ED.

## Methods

### Study design

This was a retrospective, observational study of a consecutive cohort admitted to a large urban ED in Seoul, Korea. Our Institutional Review Board approved this study, and waiver of consent was allowed because of its retrospective nature.

### Study setting and population

This study was conducted in the Department of Emergency Medicine of the Seoul St. Mary’s Hospital, a 1,320-bed tertiary teaching hospital. Our ED serves an annual census of approximately 60,000 patients. The emergency physician provides initial treatment to all adult emergency patients. We included patients older than 65 years who visited the ED with any medical problems between May 2013 and October 2013. Patients were excluded if they had a trauma-related injury; had a hematologic disease such as leukemia, myelodysplastic syndromes, myeloproliferative disease, myelofibrosis or agranulocytosis; were transferred from another hospital; were discharged from the hospital within the previous 10 days; were known to be HIV-positive; were dead on arrival or received visit-irrelevant medical treatment.

### Laboratory measurements

Blood samples for the CBC including RDW were collected when the patients were admitted to the ED, and the results were automatically stored in the clinical information system within 2 h. RDW was measured as a part of the automated CBC using the Sysmex XE-2100 (Sysmex Corp. Kobe, Japan); the reference range for RDW in our institution was 11.5 to 14.5 %. Serum chemistry and/or arterial blood gas analyses were also performed simultaneously with the CBC.

### Data collection

We abstracted the following demographic and clinical data from study participants’ medical records: age, sex, and comorbidities including cancer, diabetes, hypertension (HTN), coronary artery disease (CAD), cerebrovascular accident (CVA), congestive heart failure (CHF), chronic kidney disease (CKD) and chronic pulmonary disease. All comorbidities were defined according to the International Classification of Diseases, 10th Revision [18]. Laboratory parameters such as RDW, blood urea nitrogen (BUN), creatinine (Cr), hemoglobin (Hb), mean corpuscular volume (MCV), white blood cell (WBC), sodium, C-reactive protein (CRP), and erythrocyte sedimentation rate (ESR) were also included. A Sequential Organ Failure Assessment (SOFA) score was calculated at the time of ED admission. The primary outcome of interest was all-cause in-hospital mortality. Survival to hospital discharge was defined as discharge from the hospital alive to home or to another health care facility including a rehabilitation hospital.

### Statistical analysis

We present continuous data as means ± standard deviations (SD) for continuous variables and percentages for categorical variables. For patient characteristics and comparisons between groups, we used Student’s *t*-test for continuous variables and Fisher’s exact test and the chi-square test for categorical variables. Univariate analyses were performed to determine the predictors for all-cause in-hospital mortality. Variables with p values < 0.2 on univariate analysis were entered into the multivariate logistic regression model to create a crude model. The factors with p values < 0.05 on multivariate logistic regression model were entered into a crude model. We considered factors in a crude model as established risk factors because no confirmed risk factors exist for predicting mortality in older patients admitted to the ED.

To evaluate the association of RDW with mortality outcomes, RDW values were divided into quartiles using the following cutoff values: < 12.8 %, 12.8–13.3 %, 13.4–14.3 %, and > 14.3 %. Odds ratios (ORs) and 95 % confidence intervals (CIs) were calculated with the lowest quartile as the reference. RDW was examined as a continuous variable as well. To evaluate the prognostic value of RDW of different cutoff point, sensitivities, specificities, positive and negative predictive values, and their 95 % confidence intervals were calculated.

We estimated receiver operating characteristic (ROC) curves and compared the areas under the ROC curves (C-statistic with 95 % CI) in corresponding logistic models. All of the statistical analyses were conducted using SAS 9.3 (SAS Institute, Cary, NC, USA) and p values < 0.05 were considered statistically significant.

## Results

### Characteristics of the study population

**Fig. 1:**
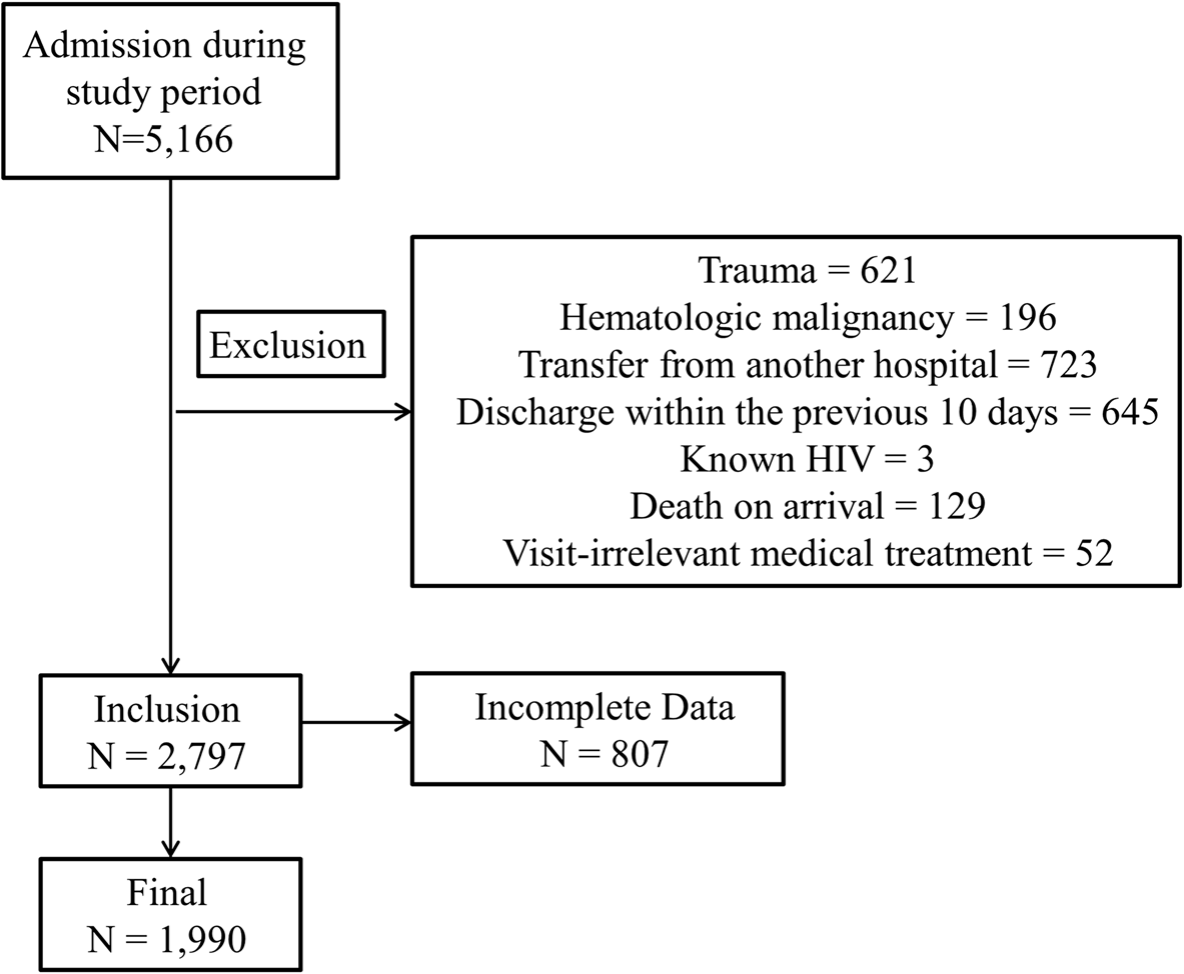
Subjects included in this study

During the study period, a total of 5,166 consecutive patients older than 65 years were admitted to our ED. Of these, 2369 patients were excluded because of trauma-related injury (*N* = 621), hematologic disease (*N* = 196), transfer from another hospital (*N* = 723), discharge from the hospital within the previous 10 days (*N* = 645), known HIV infection (*N* = 3), death on arrival (*N* = 129) and visit-irrelevant medical treatment (*N* = 52). Eight hundred seven patients were also excluded due to incomplete data. The remaining 1,990 patients were finally included in this study [Fig. 1].

The mean patient age was 75 years (SD 7), and 936 patients (47 %) were male; 74 patients (3.7 %) died during their hospital stay. The mean age was not different between survivors and non-survivors, although males were more common among non-survivors. Hypertension was the most common comorbidity. A history of cancer or chronic lung disease was more common among non-survivors. Table 1 shows the characteristics and laboratory data of the study population. The length of hospital and ICU stay was longer among non-survivors. Vasopressor and ventilator support were also more frequently required among non-survivors [Table 1].

**Table 1:** Baseline characteristics in patients with or without in-hospital mortality and comparison between patients with and without RDW values

	RDW value recorded *N* = 1990			No RDW value recorded *N* = 807			*p*
	Survivors	Non-survivors	*p*	Survivors	Non-survivors	*p*	
	*N* = 1,916	*N* = 74		*N* = 788	*N* = 19		
Demographics							
Age	74.8 ± 7.2	74.9 ± 7.6	0.931	73.8 ± 6.6	77.9 ± 8.3	0.553	0.001
Sex, male	887 (46.3)	49 (66.2)	0.001	402 (51.0)	11 (57.9)	0.008	0.047
Comorbidities							
Cancer	435 (22.7)	46 (62.2)	< 0.001	152 (19.4)	7 (36.8)	0.059	0.012
DM	614 (32.0)	18 (24.3)	0.162	247 (31.5)	5 (26.3)	0.633	0.831
HTN	1182 (61.7)	33 (44.6)	0.003	485 (61.8)	14 (73.7)	0.291	0.620
CAD	286 (14.9)	5 (6.8)	0.051	80 (10.2)	4 (21.1)	0.126	0.003
CVA	244 (12.7)	6 (8.1)	0.239	91 (11.6)	4 (21.1)	0.207	0.587
CHF	61 (3.2)	5 (6.8)	0.096	17 (2.2)	1 (5.3)	0.367	0.130
CKD	134 (7.0)	8 (10.8)	0.211	106 (13.5)	2 (10.5)	0.707	< 0.001
Chronic pulmonary disease	259 (13.5)	17 (23.0)	0.021	91 (11.6)	3 (15.8)	0.574	0.131
Laboratory Data							
BUN, mg//dl	22.5 ± 16.5	34.1 ± 20.7	< 0.001	22.2 ± 13.5	29.3 ± 16.7	0.108	0.630
Creatinine, mg/dl	1.2 ± 1.4	1.5 ± 1.0	0.023	1.3 ± 1.4	2.1 ± 3.3	0.465	0.426
Hb, mg/dl	12.6 ± 2.1	11.2 ± 2.3	< 0.001	12.6 ± 2.3	10.0 ± 2.9	0.001	0.442
MCV, fL	91.9 ± 5.6	93.3 ± 7.6	0.118	92.0 ± 6.4	91.6 ± 4.5	0.815	0.910
WBC, 10^9^/L	8.7 ± 4.1	10.6 ± 5.1	0.003	8.7 ± 10.3	14.6 ± 9.0	0.073	0.963
Sodium, mEq/L	138.5 ± 5.2	135.6 ± 7.5	0.002	139.1 ± 5.1	134.3 ± 6.0	0.004	0.092
CRP, mg/dl	3.1 ± 6.0	9.0 ± 8.2	< 0.001	2.7 ± 5.2	4.6 ± 4.0	0.273	0.097
ESR, mm/h	41.8 ± 31.4	67.9 ± 36.5	< 0.001	37.1 ± 29.8	49.1 ± 41.5	0.265	0.004
RDW, %	13.8 ± 1.7	15.9 ± 2.5	< 0.001	NA	NA	NA	< 0.001
Clinical Score							
SOFA	1.9 ± 1.6	4.4 ± 2.7	< 0.001	1.9 ± 1.4	5.7 ± 2.3	< 0.001	0.501
LOS							
Hospital	4.97 ± 9.03	14.00 ± 11.59	< 0.001	3.6 ± 6.0	6.0 ± 7.6	0.196	0.000
ICU	0.53 ± 2.45	4.00 ± 7.87	< 0.001	0.3 ± 2.0	1.5 ± 3.4	0.135	0.001
Vasopressor	46 (2.4 %)	35 (47.3 %)	< 0.001	8 (1.0)	6 (31.6)	< 0,.001	0.003
Ventilator	33 (1.7 %)	22 (29.7 %)	< 0.001	3 (0.4)	4 (21.1)	< 0,.001	0.002

*Abbreviation*: *DM* diabetes mellitus, *HTN* hypertension, *CAD* coronary artery disease, *CVA* cerebrovascular disease, *CHF* congestive heart failure, *CKD* chronic kidney disease, *BUN* blood urea nitrogen, *Hb* hemoglobin, *MCV* mean corpuscular volume, *WBC* white blood cell, *CRP* C-reactive protein, *ESR* erythrocyte sedimentation rate, *RDW* red cell distribution width, *SOFA* sequential organ failure score, *LOS* length of stay

### Logistic regression analysis

**Fig. 2:**
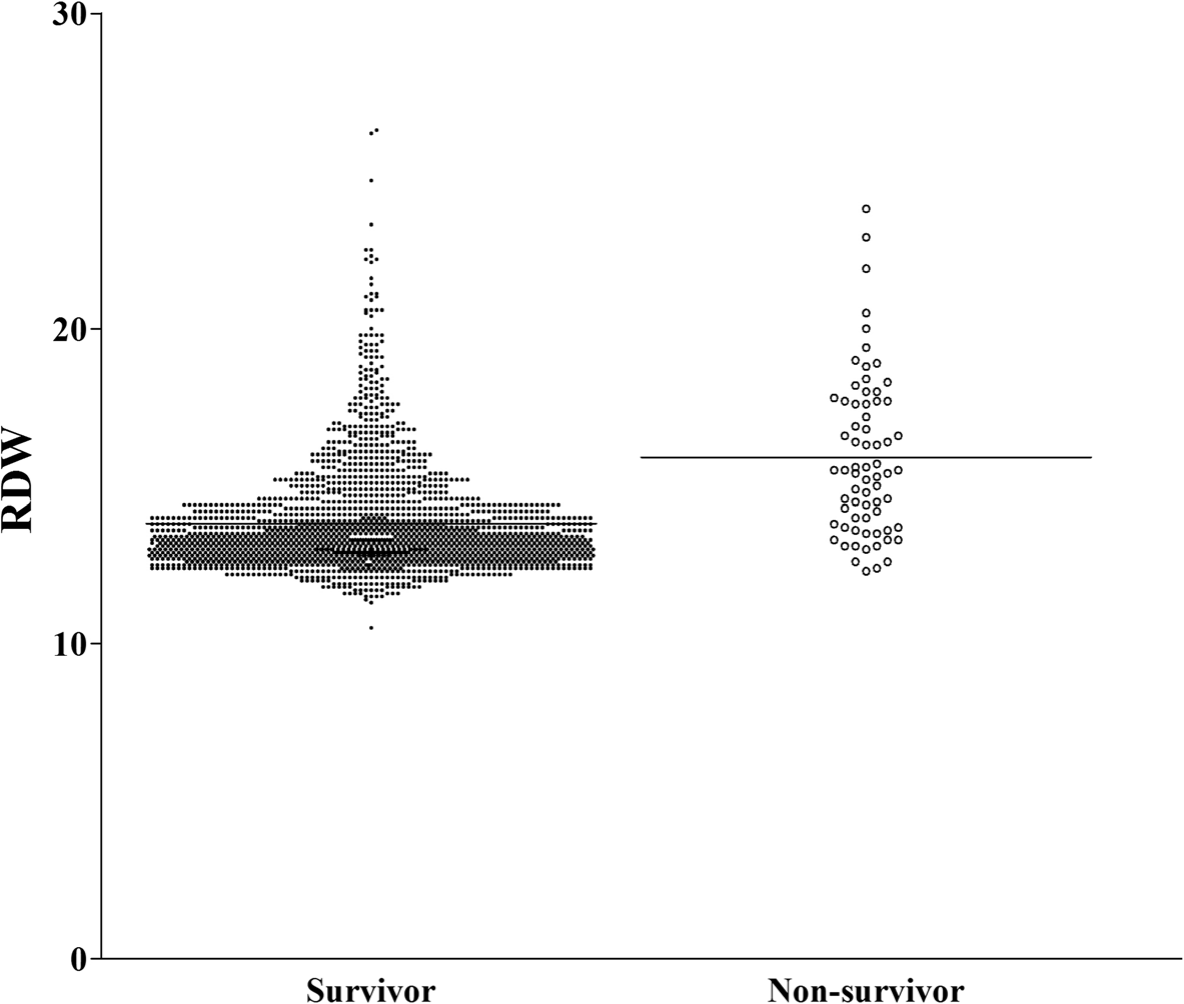
RDW according to discharge status

RDW values ranged from 10.5 to 26.3 % (mean 13.9, SD 1.8). RDW values were higher in non-survivors than in survivors (15.9 ± 2.5 vs. 13.8 ± 1.7, *p* < 0.001) [Fig. 2]. The analytical imprecision is similar between quartiles except 4^th^ quartile (coefficient of variation 2.47 for quartile1, 1.32 for quartile2, 1.82 for quartile3 and 12.2 for quartile4).

In the univariate analyses, male gender; history of cancer, HTN, and chronic pulmonary disease; and BUN, Hb, MCV, WBC, sodium, CRP, and ESR values all showed statistically significant associations with in-hospital mortality. Variables with p values < 0.2 on univariate analysis were entered into the multivariate logistic regression model to create a crude model, and the factors with p values < 0.05 in the multivariate logistic regression model were entered into a crude model. The crude model included the following variables: male gender, history of cancer, history of HTN, BUN, MCV, sodium, CRP, and ESR [Table 2].

**Table 2:** Odds ratios for all-cause in-hospital mortality events

Variables	Univariate			Multivariate		
	OR	95 % CI	*p*	OR	95 % CI	*p*
Age	1.00	0.90 – 1.03	0.931			
Sex, male	2.27	1.39 – 3.71	0.001	1.84	1.10 – 3.13	0.022
Cancer	5.59	3.46 – 9.06	< 0.001	4.21	2.51 – 7.20	< 0.001
DM	0.68	0.40 – 1.17	0.164			
HTN	0.50	0.31 – 0.80	0.004	0.48	0.28 – 0.79	0.004
CAD	0.41	0.17 – 1.03	0.059			
CVA	0.61	0.26 – 1.41	0.243			
CHF	2.21	0.86 – 5.66	0.101			
CKD	1.61	0.76 – 3.43	0.215			
Chronic pulmonary disease	1.91	1.09 – 3.33	0.023			
BUN	1.02	1.01 – 1.03	< 0.001	1.03	1.02 – 1.04	< 0.001
Creatinine	1.09	0.98 – 1.22	0.114			
Hb	0.77	0.70 – 0.85	< 0.001			
MCV	1.05	1.00 – 1.09	0.032	1.05	1.01 – 1.09	0.011
WBC	1.07	1.03 – 1.11	0.001			
Sodium	0.93	0.90 – 0.96	< 0.001	0.95	0.92 – 1.00	0.026
CRP	1.09	1.06 – 1.11	< 0.001	1.04	1.01 – 1.07	0.011
ESR	1.02	1.02 – 1.03	< 0.001	1.01	1.00 – 1.02	0.027
SOFA	1.56	1.42 – 1.72	< 0.001			
RDW	1.43	1.32 – 1.56	< 0.001			

*Abbreviation*: *OR* odds ratios, *CI* confidence interval, *DM* diabetes mellitus, *HTN* hypertension, *CAD* coronary artery disease, *CVA* cerebrovascular disease, *CHF* congestive heart failure, *CKD* chronic kidney disease, *BUN* blood urea nitrogen, *Hb* hemoglobin, *MCV* mean corpuscular volume, *WBC* white blood cell, *CRP* C-reactive protein, *ESR* erythrocyte sedimentation rate, *SOFA* sequential organ failure score, *RDW* red cell distribution width

After adjusting the crude model, RDW levels still showed an association with all-cause in-hospital mortality. Patients in the 4^th^ quartile of RDW were 5.08 times more likely to die compared with those in the lowest quartile of RDW [Model 1, Table 3]. After adjusting the crude model and SOFA score, this association remained. Furthermore, patients in the 4^th^ quartile of RDW were 3.82 times more likely to die compared with those in the lowest quartile of RDW [Model 2, Table 3].

**Table 3:** Odds ratios for RDW in the prediction of all-cause in-hospital mortality

RDW as categorical variable OR (95 % CI)					
	1^st^ quartile < 12.8 [*N* = 450, death = 4]	2^nd^ quartile 12.8–13.3 [*N* = 542, death = 8]	3^rd^ quartile 13.4–14.2 [*N* = 472, death = 10]	4^th^ quartile > 14.2 [*N* = 526, death = 52]	*p*
Model 1	1 (Reference)	1.46 (0.44–5.61)	1.83 (0.59–6.86)	5.08 (1.94–17.50)	< 0.001
Model 2	1 (Reference)	1.25 (0.36–4.97)	1.68 (0.53–6.35)	3.82 (1.42–13.38)	0.006
RDW as continuous variable OR (95 % CI)					
Model 1	1.284 (1.160–1.422)				< 0.001
Model 2	1.218 (1.095–1.355)				< 0.001
Model 1: Sex + Cancer + HTN + BUN + MCV + Na + CRP + ESR					
Model 2: Sex + Cancer + HTN + BUN + MCV + NA + CRP + ESR + SOFA					

*Abbreviation*: *RDW* red cell distribution width, *OR* odds ratios; *CI* confidence interval

When RDW was examined as a continuous variable, the mortality risk increased by 28.4 % for every 1 % increment in RDW after adjusting for Model 1 covariates (OR = 1.284, 95%CI = 1.160 - 1.422) and 21.8 % for Model 2 covariates (OR = 1.218, 95 % CI = 1.095–1.355).

### Prognostic value of RDW

Table 4 shows sensitivity, specificity, positive predictive value and negative predictive value of RDW for different cutoff point. The best cutoff value of RDW is 14.5 and has 67.6 % of sensitivity and 79.0 % of specificity.

**Table 4:** Sensitivity, specificity, positive predictive value and negative predictive value for different cutoff point

RDW	Sensitivity (95 % CI)	Specificity (95 % CI)	PPV (95 % CI)	NPV (95 % CI)
≥ 12.8	94.6 (86.7 – 98.5)	23.3 (21.4 – 25.2)	4.5 (3.6–5.7)	99.1 (97.7 – 99.8)
≥ 13.4	83.8 (73.4 – 91.3)	51.2 (48.9 – 53.4)	6.2 (4.8 – 7.9)	98.8 (97.9 – 99.4)
≥ 14.3	70.3 (58.5 – 80.3)	75.3 (73.3 – 77.2)	9.9 (7.5 – 12.8)	98.5 (97.7 – 99.1)
≥ 14.5^a^	67.6 (55.7 – 78.0)	79.0 (77.1 – 80.8)	11 (8.3 – 14.3)	98.4 (97.7 – 99.0)

*Abbreviation*: *RDW* red cell distribution width, *PPV* positive predictive value, *NPV* negative predictive value, *CI* confidence interval

^a^best cutoff value

**Fig. 3:**
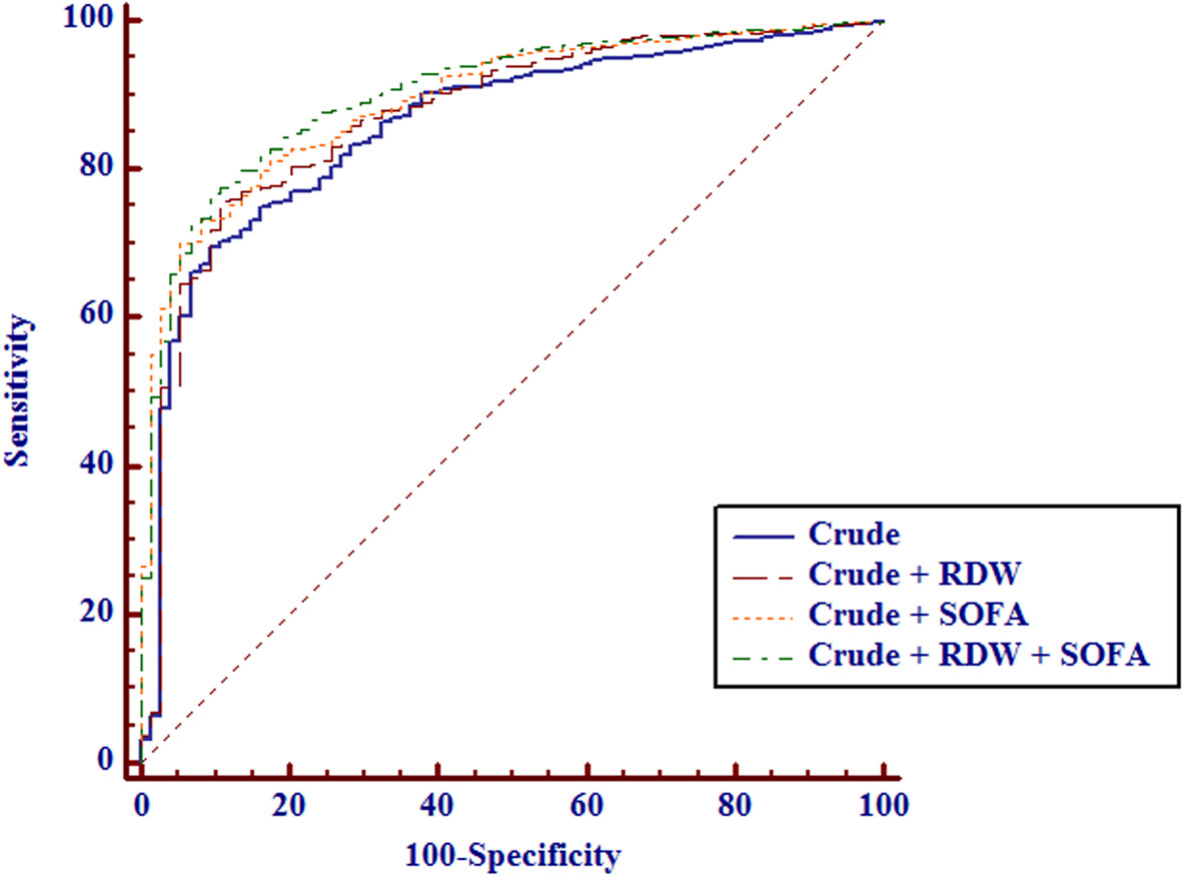
Prognostic value of RDW for the prediction of all-cause in-hospital mortality. (1) Crude model (AUC 0.858, 95 % CI 0.842–0.873); (2) Crude model with RDW (AUC 0.873, 95 % CI 0.857–0.887); (3) Crude model with SOFA (AUC 0.894, 95 % CI 0.880–0.907); (4) Crude model with RDW and SOFA (AUC 0.903, 95 % CI 0.889–0.916)

The AUC of crude model is 0.858 (95 % CI 0.842–0.873). Figure 3 shows the AUCs combined with RDW and/or SOFA in this crude model. Addition of RDW and/or SOFA to crude model improves prediction of mortality (*p* = 0.024 for RDW, *p* = 0.001 for SOFA and *p* < 0.001 for RDW and SOFA, respectively)

## Limitations

There are several limitations to this study. The first major limitation is the study’s retrospective observational design. Despite adjustment for multiple potential confounders, there may still be residual confounding factors that were unaccounted for. We also did not include data on nutrients such as vitamin B12, iron, or folate. However, these data are not routinely available in the ED. Second, this was a single-center study, which limits the generalizability of its results to the entire population of older patients admitted to the ED. Third, we could not analyze the association between RDW and cause-specific mortality because only 74 patients (3.7 %) died during their hospital stay. Therefore, more large studies are needed to prove the association between RDW and cause-specific mortality. Fourth, this study included patients older than 65 years, so these results are not applicable to young or pediatric age groups. Fifth, the physicians who were responsible for clinical decisions were not blinded the result of RDW because RDW were routinely reported in our hospital. This might be a potential source of bias and could affect the prognostic value of RDW. However, RDW was not used for clinical decisions such as hospital admission, critical procedure or discharge from hospital in our hospital. Sixth, this is a preliminary study and has a small number of patients who had the outcome of interest (*N* = 74), so the results would have to be confirmed with larger prospective cohort. Finally, there were lots of patients excluded due to incomplete data. However, we note that after adjustment for crude model i.e., male gender, history of cancer, history of HTN, BUN, MCV, sodium, CRP, and ESR there was no difference in in-hospital mortality between patients included and excluded in this study cohort. After adjusting the crude model and SOFA score, this association remained.

## Discussion

In this study, we found that the RDW value at admission was an independent predictor of all-cause in-hospital mortality among patients older than 65 years. Indeed, the RDW values were higher in non-survivors than in survivors. After adjustment for male gender, history of cancer and HTN, levels of BUN, MCV, sodium, CRP, and ESR and SOFA scores, the all-cause in-hospital mortality rate increased by 21.8 % for each 1 % increase in RDW as a continuous variable. These findings are consistent with other recently published studies [16, 17]. When using the best cutoff value of RDW, the PPV and NPV are 11 % (95 % C.I. 8.3 – 14.3) and 98.4 % (95 % C.I. 97.7 – 99.0), respectively. If a patient has RDW of greater than 14.5, the risk of in-hospital death is 11 %. If a patient has a RDW of smaller than 14.5, the risk of survival hospital discharge is 98.4 %.

The elderly population is increasing and presents significant challenges to the health care system. Elderly patients tend to visit the ED more frequently than young adults, often present with atypical signs and symptoms, and have multiple comorbidities that complicate accurate diagnosis, treatment and mortality prediction [5]. As a result, older patients have a higher risk of in-hospital mortality than younger patients [5], and most emergency physicians feel less comfortable when treating older patients than their younger counterparts [19]. Clinicians also realize that complete assessments of older patients are difficult and time consuming. Because of these considerations, Samaras et al. proposed a targeted approach or high-risk patients [4]. Our results may also aid in the identification of older patients at the highest risk.

The exact mechanisms for the association between RDW and mortality are not well understood. However, an increased RDW is associated with the extent of systemic inflammation, and elevated biomarkers of inflammation such as ESR, interleukin and CRP levels are associated with an elevated RDW [20, 21]. Inflammation suppresses bone marrow function, and inflammatory cytokines can suppress erythrocyte maturation, which in turn may lead to an elevated RDW. Increased RDW has also demonstrated an association with oxidative stress and activation of the renin-angiotensin-aldosterone system [22, 23].

RDW has been regarded as a potential predictor of mortality in clinically significant cardiovascular disease, stroke, septic shock, bacteremia and community-acquired pneumonia [10–14]. In addition, several recent studies have reported that RDW is predictive of all-cause mortality in critically ill or ICU patients [15, 24, 25]. Our study provides novel evidence regarding the prognostic value of RDW in older patients admitted to the ED, which suggests that RDW may serve as an early prognostic marker for the entire spectrum of acute disease, rather than confined to specific conditions, in these patients. Thus, a high RDW level at initial presentation to the ED among older patients may provide evidence to aid the decision-making process regarding admission to the hospital regardless of etiology. However, this study is generating hypothesis rather than confirming. So, large prospective studies are needed to prove and validate this hypothesis.

This study raises a few questions that should be verified in the future. As discussed before, the exact mechanisms that underlie the association between RDW and mortality are still unknown. And how to use RDW in real clinical setting is still unknown. Even though several studies have proven that RDW is a potential predictor of mortality in various critical diseases, little is known that RDW could guide treatment or support clinical decision. The time course of RDW would provide more valuable information to clinicians. RDW trend over time would be useful to assess the severity of disease or guide therapy.

Prediction of in-hospital mortality or risk stratification in patients presenting to the ED is challenging and complex. Several scoring systems have been developed, although none of them are perfect and external validation has not been fully conducted [26]. Moreover, prediction of in-hospital mortality among older patients admitted to the ED has rarely been investigated. Most of the current scoring systems use vital signs and level of consciousness as variables when calculating the patient’s score. Our study did not include vital signs or level of consciousness as predictors of in-hospital mortality; however, this omission was not considered a limitation of this study because we included a SOFA score that was based on six different scores, one each for the respiratory, cardiovascular, hepatic, coagulation, renal and neurological systems [27]. Moreover, the SOFA score includes mean arterial pressure and the Glasgow Coma Scale. And older adults admitted to the ED are likely to have multiple comorbidities. This raises the concern that the patients with high RDW have a potential to have multiple or severe comorbidities. According to the result of multivariate logistic regression analysis, in-hospital mortality in our cohort was associated with RDW rather than multiple comorbidities. However, this should be further tested.

## Conclusions

In conclusion, RDW at admission to the ED is associated with all-cause in-hospital mortality among patients older than 65 years. Thus, RDW values may serve as a surrogate marker of disease severity. We caution against using these findings to aid clinical decision-making process until they are externally validated.

## Abbreviations

AUC, area under the curve; BUN, blood urea nitrogen; CAD, coronary artery disease; CBC, complete blood count; CHF, congestive heart failure; CKD, chronic kidney disease; Cr, creatinine; CRP, C-reactive protein; CVA, cerebrovascular accident; ED, emergency department; ESR, erythrocyte sedimentation rate; Hb, hemoglobin; HTN, hypertension; ICU, intensive care unit; MCV, mean corpuscular volume; RDW, red cell distribution width; ROC, receiver operating characteristic; SOFA, Sequential Organ Failure Assessment; WBC, white blood cell
